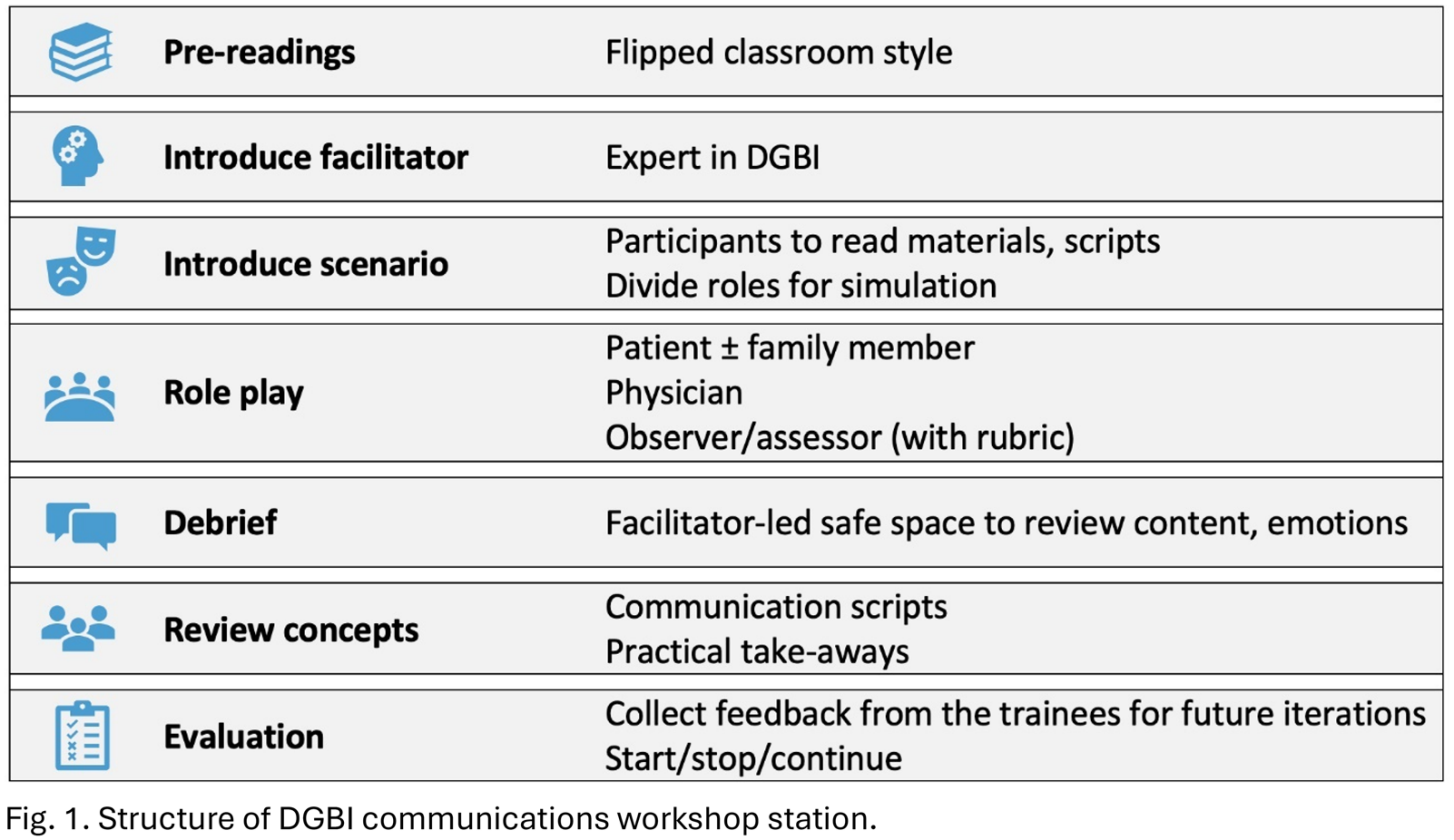# Poster Session I - A121 DEVELOPMENT OF A COMMUNICATIONS CURRICULUM FOR GASTROENTEROLOGY TRAINEES: A PILOT STUDY

**DOI:** 10.1093/jcag/gwaf042.121

**Published:** 2026-02-13

**Authors:** G Wang, C Gafrey, C Parker, C M Walsh, L Targownik

**Affiliations:** Gastroenterology, University of Toronto, Toronto, ON, Canada; Medicine, University of Toronto, Toronto, ON, Canada; Gastroenterology, University of Toronto, Toronto, ON, Canada; Gastroenterology, University of Toronto, Toronto, ON, Canada; Gastroenterology, University of Toronto, Toronto, ON, Canada

## Abstract

**Background:**

Effective communication is essential for gastroenterology (GI) patient-provider rapport and outcomes, yet trainees receive little formal instruction in these skills. Patients with Disorders of Gut-Brain Interaction (DGBI), who make up a substantial proportion of GI practice, benefit most from empathetic communication, yet training in these approaches is nearly absent. In addition, certain marginalized populations gain disproportionate benefit from respectful and inclusive language. While research shows that communications curricula improve provider competence and patient satisfaction, such programs are virtually nonexistent in GI, representing a critical gap that directly affects patient care and equity.

**Aims:**

To develop a workshop curriculum teaching practical communication skills to GI trainees. Our pilot included a station on DGBI and a station on GI health for Indigenous patients. The curriculum emphasized building a toolbox of scripts and practical counselling strategies.

**Methods:**

We consulted content experts in DGBI, Indigenous health, and medical education to construct workshop stations. We developed pre- and post-surveys, adapted from validated questionnaires, to assess trainee knowledge and confidence. We will calculate mean scores using descriptive statistics and evaluate changes in knowledge and confidence from matched pre- and post-surveys.

**Results:**

We developed two pilot workshop stations: communication with patients with DGBI (Fig. 1) and GI health for Indigenous patients. Each station incorporated flipped classroom-style pre-readings, facilitator-led didactic teaching with defined objectives, simulation role play with peer assessment, facilitator-led debriefing with practical takeaways, and trainee feedback to guide improvement.

The DGBI station featured a patient with IBS, requiring sensitive counselling about their diagnosis and management. It emphasized the stigma and frustration often experienced by patients with DGBI, and provided concrete scripts and approaches to foster empathy, validate symptoms, and strengthen rapport when symptoms cannot be “fixed”.

The Indigenous health station addressed the impact of historical discrimination and provider inexperience on patient trust. Developed collaboratively with Indigenous community partners, it integrated teachings about traditional diets, medicines, and connections to the land that support GI health, and introduced local resources, translation services, and support networks to promote culturally safe care.

**Conclusions:**

We plan to iteratively refine and expand this workshop based on trainee feedback. We hope that this curriculum equips GI physicians to strengthen therapeutic relationships, improve treatment adherence and patient satisfaction, manage expectations, reduce unnecessary healthcare utilization and patient complaints, and ultimately improve GI health outcomes.

**Funding Agencies:**

University of Toronto Department of Gastroenterology Research Innovation Grant